# Childhood unpredictability and the development of exploration

**DOI:** 10.1073/pnas.2303869120

**Published:** 2023-11-27

**Authors:** Yuyan Xu, Madeline B. Harms, C. Shawn Green, Robert C. Wilson, Seth D. Pollak

**Affiliations:** ^a^Department of Psychology, University of Wisconsin–Madison, Madison, WI 53706; ^b^Department of Psychology, University of Minnesota Duluth, Duluth, MN 55812; ^c^Department of Psychology, University of Arizona, Tucson, AZ 85721; ^d^Cognitive Science Program, University of Arizona, Tucson, AZ 85716

**Keywords:** unpredictability, exploration, decision-making, development, cognitive flexibility

## Abstract

Early in development, the process of exploration helps children gather new information that fosters learning about the world. Yet, it is unclear how childhood experiences may influence the way humans approach new learning. What influences decisions to exploit known, familiar options versus trying a novel alternative? We found that childhood unpredictability, characterized by unpredictable caregiving and unstable living environments, was associated with reduced exploratory behavior. This effect holds while controlling for individual differences, including anxiety and stress. Individuals who perceived their childhoods as unpredictable explored less and were instead more likely to repeat previous choices (habitual responding). They were also more sensitive to uncertainty than to potential rewards, even when the familiar options yielded lower rewards. We examined these effects across multiple task contexts and via both in-person (N = 78) and online replication (N = 84) studies among 10- to 13-y-olds. Results are discussed in terms of the potential cascading effects of unpredictable environments on the development of decision-making and the effects of early experience on subsequent learning.

## Significance

Exploration, or sampling for new information, facilitates discovery and socio-emotional learning that sets the stage for adaptation and well-being later in life, yet it is costly in the short run. In this experiment, children who experienced their lives as less predictable explored less for information because of a preference for familiarity and a tendency to repeat their previous responses—even when those choices yielded lower rewards. Thus, this study revealed that exploration could be a pivotal learning parameter that influences developmental trajectories and that environmental unpredictability, though currently understudied, constitutes an important dimension of the childhood environment and warrants greater attention in understanding human development.

Decision-making often requires us to choose between selecting options that are familiar and that have resulted in positive outcomes in the past (exploitation) versus trying something new that could result in either a better or a worse outcome than the familiar option (exploration). As an example, a favorite restaurant might present a choice between selecting a familiar dish that has been consistently quite good in the past versus trying a new special offering that might (or might not) be even more pleasing. Early in development, these types of decisions about whether to actively sample and explore new features of the world are a critical component of how humans learn. In general, children are more likely than adults to try new options and consider novel hypotheses (1–3). Yet, this tendency is not independent of the environment that children find themselves within. For example, environmental predictability facilitates learning by allowing children to form expectations based on their experiences (4, 5). In essence, the presence of some degree of predictability means that there is value in attempting to find relations, structure, or interesting outcomes in the world because if these are learned, they could then later be exploited. Might less stable environments discourage children from exploring? We examine this question by testing the effects of chronic early childhood unpredictability on children’s decisions to explore.

Exploration constitutes an important learning mechanism in childhood. Adaptive decision-making requires that children recognize probabilistic associations between cues and outcomes. For example, children learn what to eat and touch based on their bodily reactions, and they learn how to navigate the social world by inferring others' mental states from their behaviors (6, 7). Children engage in more exploration than adults in social learning and reward-gathering contexts (8–11). These tendencies to explore not only allow children to sample a wider variety of data to inform their behaviors but also foster adaptiveness and flexibility in learning (12, 13). There is some evidence that childhood experience influences exploratory behaviors. When feeling safe in the presence of caregivers, young children will approach novel objects, even when they are aversive (14). On the contrary, after experiencing unstable caregiving in orphanages, children tend to exploit options with known rewards regardless of the context (15). Thus, predictable environments appear to facilitate learning by encouraging exploration and discovery.

Research on childhood adversity generally relies on methods that focus on events in children’s lives. Yet, recent evidence suggests that how individual children perceive, interpret, or understand the events in their lives may hold the key to understanding these phenomena (16, 17). What might lead a child to perceive their environment as unpredictable? Chronic experiences with adults who fail to fulfill promises, follow through with planned activities, control their emotions and behaviors, or use consistent disciplinary techniques may lead children to view the interpersonal world as unstable (18). Similarly, frequent or unexpected changes of parental custody, unstable housing, and fluctuations in family income can undermine a sense of predictability and alter behavioral and cognitive functions (19–22). Consistent with this view, infant exposure to less-predictable maternal sensory signals results in subsequent impairments in cognitive development in both humans and rodents (23, 24). Still unknown, however, is whether children’s perceptions of the predictability of their environments influence their tendency to explore in search of rewards.

Childhood unpredictability may influence exploration through different mediating mechanisms. First, unpredictable environments may trigger increased temporal discounting, a tendency to maximize immediate rewards at the cost of long-term gains or strategic exploration. For example, individuals who experience resource scarcity in childhood approach temptations more quickly (25). Even among children without adverse experiences, brief interactions with unreliable people who fail to keep promises trigger more impulsive acts to favor immediate over delayed rewards (26, 27). Second, unpredictable childhood experiences may lead individuals to consistently resort to habits or default options as a way to minimize uncertainty (28). Children who endure high levels of adversity show inflexibility in learning and decision-making (29–32). This inflexibility appears to lead to a bias for inaction over initiating new actions (33–35).

**Fig. 1.**
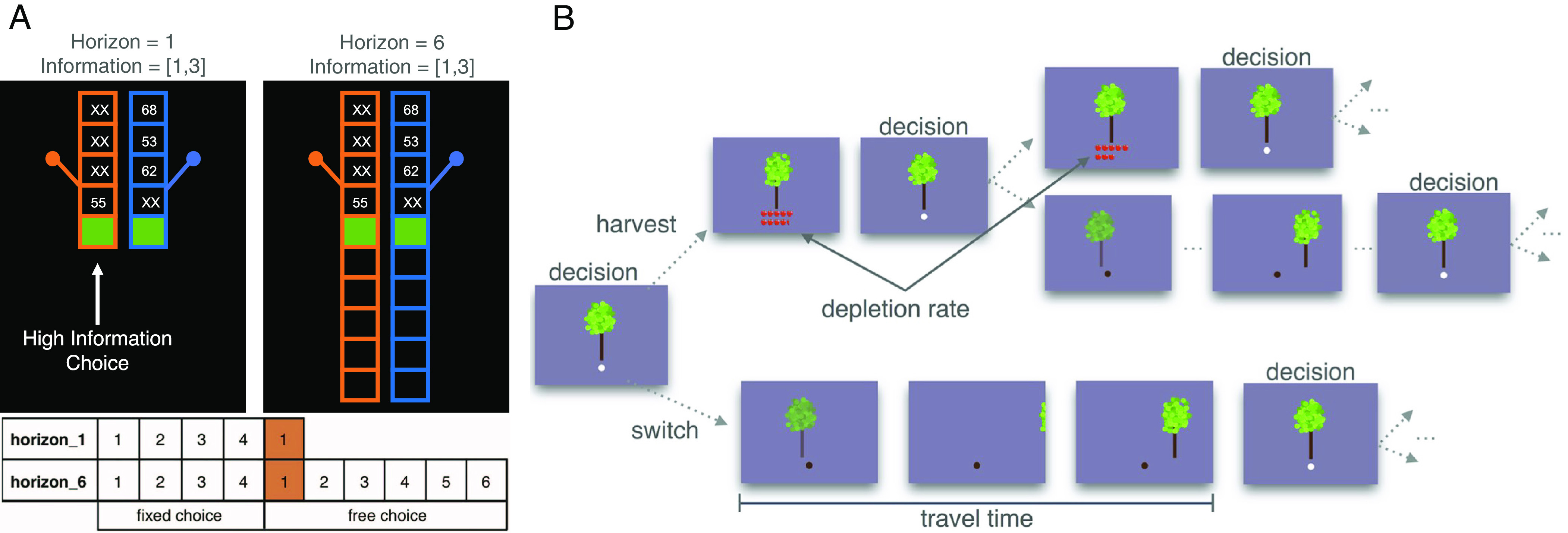
Task design. (*A*) In the Horizon task, participants chose between two bandits whose payout values are hidden. Within the first four choices, the computer revealed the payouts three times for one bandit and once for the other bandit, creating an “unequal information” condition. Information-directed exploration was defined as selecting the bandit with one payout (more informative) at the first free choice. The length of each game was manipulated to have either one or six free choices, rendering exploration more advantageous in long-horizon games. Figure adapted from Somerville et al. (37). (*B*) In the Orchard task, participants decided whether to harvest at the current tree, where apples depleted over time, or switch to the next tree with a full supply of apples. Exploration was measured by exit thresholds, the average of the last two harvests before moving to a different tree. A high exit threshold indicates that participants moved to the next option faster and thus represents a higher exploration rate. The travel time (short or long) indicated the cost of switching in different orchards, representing the “rich” and “poor” foraging environment, respectively. Figure adapted from Lenow et al. (34).

Here, we examined whether and how unpredictable childhood environments influence exploration. To do so, we tested 10- to 13-y-olds on exploration tasks that require information seeking and foraging, respectively. One task assessed information-seeking under uncertainty, whereas the second task required balancing the trade-off between immediate rewards and expected future rewards (36; Fig. 1). We chose this age range because it is a time of increasing exposure to novel social environments (e.g., transitioning from elementary to middle school, forming new peer groups) that offers opportunities to explore. Exploratory behaviors in the social domain become highly active at this stage compared to younger children and adults (9). Individuals in this age range also begin to exhibit more strategic use of exploration (37), as this is the developmental period when executive processes such as cognitive flexibility undergo substantial development (38) and adult-like performance on reward-driven tasks is competent but not yet at adult levels (39, 40). We examine one a priori and two exploratory hypotheses. First, we predicted that children who perceive their environments as unpredictable would engage in less exploration than peers who perceive themselves as living in relative stability. If this hypothesis was confirmed, we sought to examine two ancillary issues. The first was whether temporal discounting (preferring short-term rewards over information for future uses) or habitual responding (persisting with previous behaviors even when they become suboptimal) were mechanisms that could account for less exploratory behavior. Of note, these mechanisms could be mutually exclusive in scenarios when an uncertain option might yield a high one-time reward. Here, temporal discounting would predict approaching behavior, whereas habitual responding would predict avoidance. Because prior studies often conflate reward and uncertainty (15), we sought to disentangle these factors. Second, we examined potential differences in variations in exploration such as information-seeking versus foraging behavior. Finally, we conducted a replication study prior to reporting these data. To address the possibility that related individual differences other than unpredictability might account for children’s behavior, we controlled for anxiety and subjective stress in the Primary and Replication studies, respectively.

## Results

### Information Gathering.

#### Baseline evidence of exploration.

We first examined whether this task elicits exploration behaviors and whether the task manipulations (i.e., uncertainty, rewards, and horizon) were effective. Children chose the more informative option in both short- and long-horizon games even when it had a lower mean payout history. In addition, children showed a strategic use of exploration—they explored more in long-horizon games than in short-horizon games (Table 1).

**Table 1.** Baseline exploration in the Horizon task by study

	Primary study				Replication study			
	*t*	*df*	*P*	95% CI	*t*	*df*	*P*	95% CI
Exploration in short horizon games	52.81	77	<0.001	[4.99, 6.93]	38.67	83	<0.001	[3.54, 4.88]
Exploration in long-horizon games	41.98	77	<0.001	[3.97, 5.52]	36.51	83	<0.001	[3.34, 4.61]
Strategic exploration	−2.15	154	0.02	[−0.66, −0.03]	−1.79	166	0.04	[−0.58, 0.03]

Note: One-sample *t* tests were used to examine whether children explored in short- and long-horizon games, respectively. A two-sample *t* test (one-tailed) was used to examine whether children explored more in long-horizon games than in short-horizon games. *df* = Degree of freedom; CI = Confidence interval.

#### Is childhood unpredictability associated with reduced exploration?

**Fig. 2.**
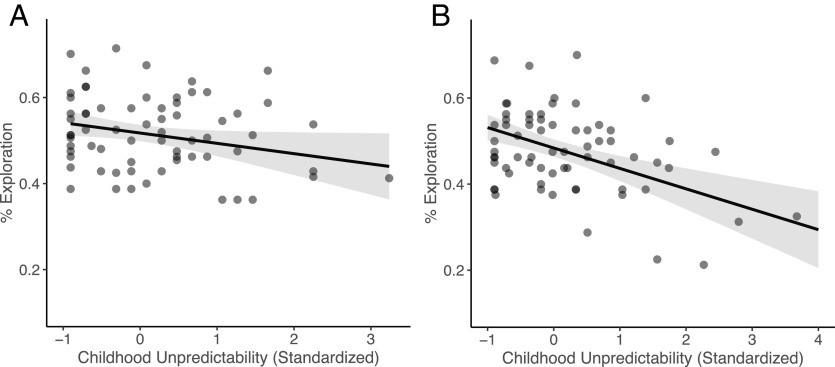
In the Horizon task, children who reported more childhood unpredictability explored less in both the Primary (*A*) and Replication (*B*) studies (*P* = 0.04; *P* < 0.001). 95% CIs are plotted.

To test whether childhood unpredictability is associated with reduced exploration, we regressed the average rate of choosing the more informative option at the first free choice on childhood unpredictability. Children who reported more childhood unpredictability were less likely to choose the informative option [Primary study: B = −0.02, SE = 0.01, *t*(73) = −2.08, *P* = 0.04; Replication study: B = −0.05, SE = 0.01, *t*(76) = −4.32, *P* < 0.001; Fig. 2 *A* and *B*].

**Fig. 3.**
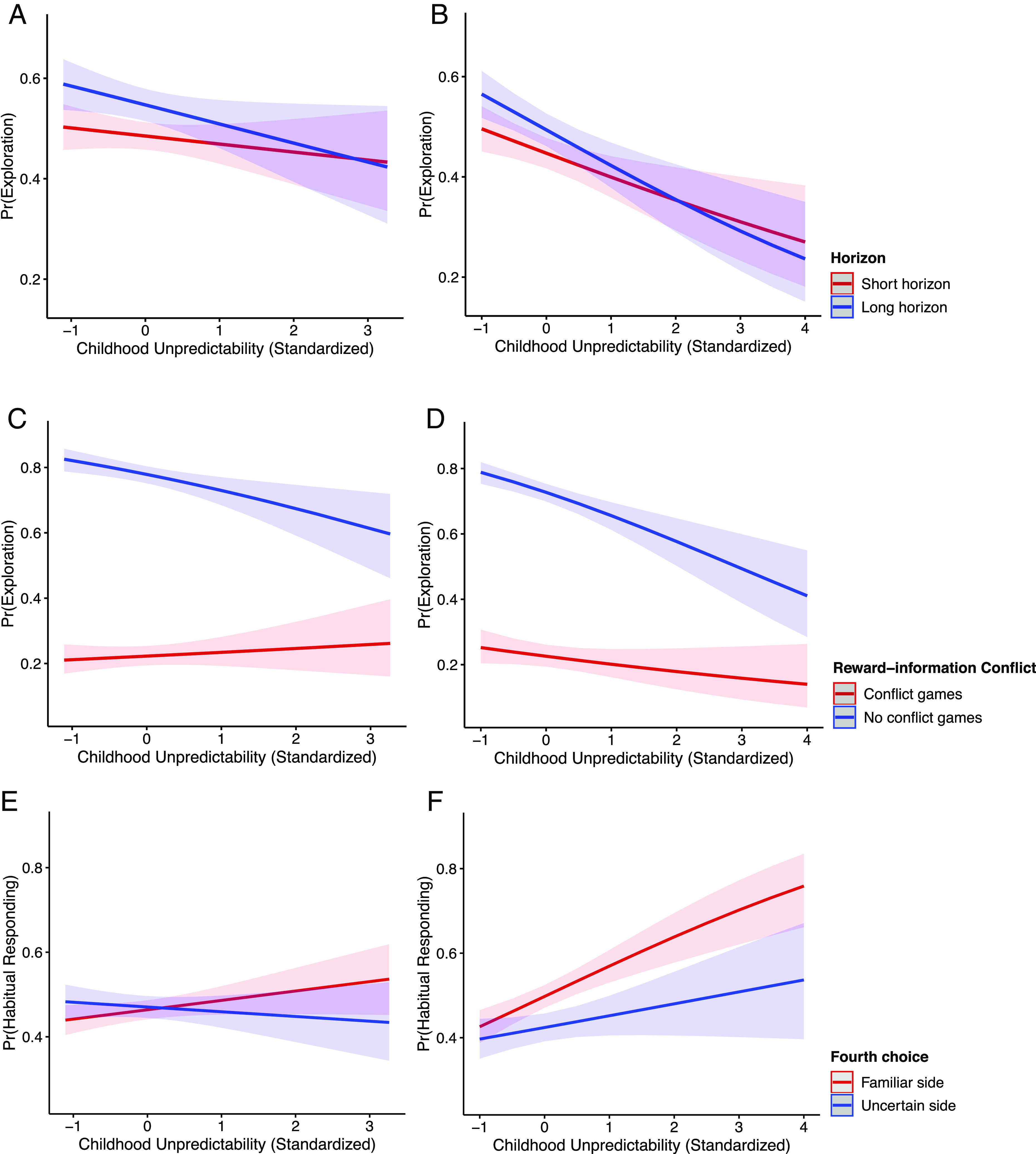
Relationships between childhood unpredictability and explore-exploit choices differ by task conditions in the Horizon task. (*A* and *B*) Strategic exploration. Children who reported more childhood unpredictability used less strategic exploration in both the Primary (*A*) and Replication (*B*) studies (*P* = 0.03; *P* = 0.05). They were less likely to choose the informative option, especially in long-horizon games where that information could be used to guide future choices. (*C* and *D*) Temporal discounting. Children who reported more childhood unpredictability did not demonstrate increased temporal discounting, as they were less likely to adjust their information seeking across conflict and no-conflict games in both the Primary (*C*) and Replication (*D*) studies (*P* = 0.002; *P* = 0.04). There was no evidence from either study that those who reported high unpredictability explored less in reward-information conflict games or that they explored more in no-conflict games where the high informative option yields more rewards. (*E* and *F*) Habitual responding. Children who reported more childhood unpredictability showed an increased preference for familiar options over exploring uncertain options in both the Primary (*E*) and Replication (*F*) studies (*P* = 0.07; *P* = 0.02). 95% CIs are plotted.

We then examined whether the strategic use of exploration varied with childhood unpredictability. Estimating a generalized mixed-effects model using trial-by-trial data, we regressed the first free choice on an interaction between childhood unpredictability and horizon. Childhood unpredictability was associated with less strategic information seeking (Primary study: Odds Ratio = 0.82, *P* = 0.03, 95% CI: [0.68, 0.98]; Replication study: Odds Ratio = 0.83, *P* = 0.05, 95% CI: [0.68, 1.00]; Fig. 3 *A* and *B*). Estimated marginal effects suggested that children who reported more childhood unpredictability were less likely to choose the informative option, especially in long-horizon games where that information could be used to guide future choices (estimated marginal effects of childhood unpredictability on exploration in long-horizon games: Primary study: M = −0.14, SE = 0.07, CI = [−0.29, −0.002]; Replication study: M = −0.28, SE = 0.07, CI = [−0.42, −0.15]).

#### Is this effect specific to perceived unpredictability?

In addition to children’s reports, we also assessed stressful and negative events in our participants’ lives from their parents. We did not find evidence that parent reports of adverse or negative life events predicted changes in information seeking [B = 0.002, SE = 0.003, *t*(72) = 0.81, *P* = 0.43] or strategic exploration (Odds Ratio = 0.98, *P* = 0.61, 95% CI: [0.93, 1.04]). We also examined the possibility that the effects we observed on exploration could be explained merely by some children having a predisposition to perceive or report everything as generally unstable. However, this explanation was not supported by the data. For example, we observed no significant correlation between children’s assessments of the stability of food and money and that of parental monitoring in the Questionnaire of Unpredictability in Childhood (QUIC) [Pearson correlation coefficient in the Primary study: *r*(76) = 0.15, *P* = 0.18; Replication study: *r*(82) = 0.10, *P* = 0.38; *SI Appendix*, Table S3 and S4]. Moreover, individual variability plots indicate that children who reported high unpredictability tended to rate only one aspect of their childhood as relatively highly unstable (*SI Appendix*, Fig. S3), suggesting that these ratings were specific instead of reflective of children’s general traits or response styles. Overall, these data are consistent with an influence of childhood unpredictability on exploration behaviors as well as highlighting the importance of considering children’s own perspectives on their life experiences (17).

#### What accounts for the relationship between childhood unpredictability and reduced exploration?

To test whether children who experienced more unpredictability tended to prioritize rewards over information (temporal discounting), we estimated a generalized mixed-effects model using trial-by-trial data and regressed the first free choice on an interaction between childhood unpredictability and a binary variable encoding whether the more informative option was the more rewarding option (i.e., whether the choice presents a reward–information conflict). Results did not support the temporal discounting explanation, as children with higher levels of unpredictability were less likely to adjust their information-seeking across conflict and no-conflict games (Primary study: Odds Ratio = 0.65, *P* = 0.002, 95% CI: [0.49, 0.85]; Replication study: Odds Ratio = 0.75, *P* = 0.04, 95% CI: [0.57, 0.99]; Fig. 3 *C* and *D*). Specifically, in the reward-information conflict trials, children with more unpredictability did not favor the rewarding option more than the informative option (estimated marginal effects of childhood unpredictability on exploration in conflict games: Primary study: M = 0.06, SE = 0.09, CI = [−0.12, 0.25]; Replication study: M = −0.15, SE = 0.10, CI = [−0.34, 0.05]). Even when the more informative option yielded more rewards, these children did not explore more (estimated marginal effects of childhood unpredictability on exploration in no-conflict games: Primary study: M = −0.26, SE = 0.08, CI = [−0.43, −0.10]; Replication study: M = −0.34, SE = 0.07, CI = [−0.47, −0.20]). Thus, childhood unpredictability did not appear to orient children to immediate rewards over information.

**Fig. 4.**
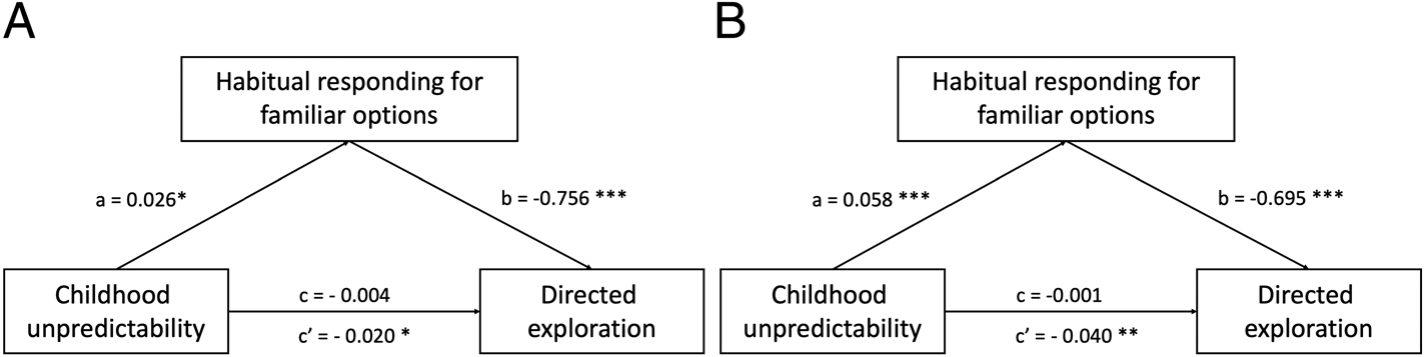
In the Horizon task, the relationship between childhood unpredictability and reduced exploration was mediated by habitual responding in both the Primary (*A*) and Replication (*B*) studies. *P < 0.05, **P < 0.01, ***P < 0.001.

We next tested whether childhood unpredictability was associated with children’s preference for familiar options (habitual responding). We estimated a generalized mixed-effects model where choice uncertainty (whether the option has more or less available information) interacted with childhood unpredictability to predict habitual responding (whether children chose the previous option at the first free choice). Results supported this mechanism (Primary study: Odds Ratio = 0.87, *P* = 0.07, 95% CI: [0.76, 1.01]); Replication study: Odds Ratio = 0.84, *P* = 0.02, 95% CI: [0.72, 0.97]; Fig. 3 *E* and *F*). Children who reported more childhood unpredictability were more likely to repeat their previous choice if it was the less uncertain option, regardless of its reward values (estimated marginal effects of childhood unpredictability on habitual responding following an uncertain option: Primary study: M = 0.08, SE = 0.05, CI = [−0.01, 0.19]; Replication study: M = 0.29, SE = 0.06, CI = [0.17, 0.40]). This analysis provided preliminary evidence that childhood unpredictability selectively primed habitual, repetitive responses to familiar options. Post hoc analyses confirmed that habitually choosing familiar options mediated the relationship between childhood unpredictability and reduced exploration (Fig. 4 *A* and *B*). There were no differences in children’s reaction time or reward maximization behaviors associated with childhood unpredictability (*SI Appendix*).

### Foraging.

We ran a linear mixed-effects model regressing trial-level exit thresholds on the interaction between childhood unpredictability and environment type (long or short travel time). First, as a manipulation check, we looked at the main effect of environment type. Children left the current tree earlier in short-travel-time (“richer”) orchards and later in long-travel-time (“poorer”) orchards (B = 0.91, SE = 0.14, *t* = 6.53, *P* < 0.001), confirming that the task manipulation was successful. However, no evidence emerged associating childhood unpredictability with patch-switching behaviors (exploration; main effect of childhood unpredictability: B = 0.02, SE = 0.27, *t* = 0.08, *P* = 0.94; interaction between childhood unpredictability and environment type: B = −0.05, SE = 0.15, *t* = −0.33, *P* = 0.74). We then examined how much children’s exploration behaviors deviated from optimal strategies. One-sample *t* tests showed that overall, children explored more than optimal in both short-travel-time orchards [*t*(75) = 6.09, *P* < 0.001] and long-travel-time orchards [*t*(75) = 5.89, *P* < 0.001].

## Discussion

Lack of environmental predictability is an important yet understudied component of adversity in childhood. That children who perceived and experienced their lives as unpredictable engaged in less exploration offers new insight into how early experiences shape subsequent parameters of human learning. In the present experiments, children who experienced their lives as less predictable explored less, which was accounted for by a preference for familiarity and a tendency to repeat their previous responses—even when those choices yielded lower rewards. This effect holds while controlling for individual differences such as anxiety or subjective stress. This sense of environmental unpredictability appears to affect both cognitive flexibility as well as prime individuals to be more sensitive to uncertainty than to potential rewards. Yet, unpredictability only affected children’s information-seeking and not their foraging behavior, suggesting some specificity in the effects of this experience.

Adaptive learning requires a balance of strategies. Depending on the situation, searching either broadly or within a narrow range may be optimal; sometimes it is best to harness flexibility and consider new options, whereas at other times maintaining stability will lead to the best outcomes. Another distinction is that while exploration tends to pay off in the long run, affording more opportunities for discovery, it is often more costly and time-consuming in the short run (41). It makes sense that humans appear to engage in more exploration early in development and then shift to more exploitation with greater maturity (42): Sampling for information helps uncover cue–outcome associations that allow children to better learn how to navigate their social and physical worlds. Decreased exploration implies fewer opportunities to learn about environments and the people in them, which can be reflected in behavior choices (43). In this regard, the present findings shed light on active learning and information sampling as a potential cognitive mechanism through which childhood experiences influence developmental outcomes.

Why might environments that children perceive as less predictable affect these learning processes? There are several related and nonexclusive possibilities. One is that the factors that facilitate exploration and discovery in children—curiosity, playfulness, positive affect, motivation, and wider attention (42)—may be attenuated by the same issues that undermine stability in children’s environments. Chronic instability may lead children to perceive that exploration confers too much risk, leaving these individuals fewer opportunities for practice engaging in this strategy. A related possibility is that children in less predictable environments may not perceive themselves as having the resources to widely explore; if resources or support are thin, a good-enough solution might seem a better choice than searching for a possible better solution. The adversity conferred by unpredictability could lead children to become risk averse (15). Indeed, unpredictable stress in rodents leads to increases in rates of exploitation over exploration (44). Finally, unpredictability, like other dimensions of childhood adversity, may trigger an early transition to adult-like neural and behavioral patterns as an adaptation to adverse environments (stress acceleration theory; 45, 46), leading to a developmental shift from exploration to exploitation.

One possible alternative interpretation of these findings is that some children may have traits that leave them to perceive or report everything as unstable. However, this possibility was not supported by our data. Child-rated unpredictability uniquely explains changes in exploratory behavior beyond any other source of individual differences we measured that would suggest the effects reflect a child’s trait. In addition, children’s assessments of the stability of some domains of their lives were uncorrelated. We also assessed so-called “objective” stressful and negative events in our participants’ lives by asking their parents about them. Yet, it was only children’s subjective sense of whether their lives were unpredictable that accounted for reductions in exploration behaviors (16, 17).

Three features of children’s behavior uncovered in these experiments are likely to lead to fruitful future discoveries. First, when children from more unpredictable environments explored, they tended to do so less strategically (e.g., by foregoing the informative option in long-horizon games), which is consistent with prior evidence that unpredictability is linked to reduced future orientation and attenuated inhibitory control (20, 22, 23, 47–49). It is also consistent with the idea that variations in learning environments are conducive to different types of decision-making strategies. For example, children who experience more unpredictability expect more volatility and less stability in their environments.

Second, children who experienced more unpredictability tended to repeat responses that they used previously and were more familiar with, even if those options yielded lower rewards. This pattern suggests inflexible decision-making, driven by an increased avoidance of uncertainty that potentially compromises reward sensitivity. This observation could also reflect difficulties in learning or using the value of information to guide future choices (50). Prior research found attenuated reward sensitivity following childhood adversity (51, 52), but questions remain about how uncertainty interacts with reward processing to shape decisions (53)—for example, whether the effect of childhood unpredictability on goal-directed learning and decision-making is more salient under heightened uncertainty.

Third, the observation that unpredictability affected exploration in the context of information seeking, but not foraging, also suggests that heightened uncertainty may be playing a critical role. In the Horizon task, uncertainty is constantly present: Participants must resample every time new bandits are presented. In contrast, uncertainty peaks as participants in the Orchard task enter a new field but soon diminishes because every patch in that orchard carries the same information (i.e., average reward and depletion rates). Yet, this hypothesis was not in the scope of the current experiments. One reason is that the current Horizon task did not have a condition where both bandits offered equal information before the first free choice. Including this condition in future studies would allow the possibility of examining the effect of childhood unpredictability on random exploration in addition to the strategic exploration examined here. Another is that because the Orchard task did not manipulate uncertainty (i.e., the reward and depletion rates are stable in each orchard), we cannot conclude that the lack of an effect of childhood unpredictability reflects something different about foraging. Yet, this absence of evidence does not necessarily negate the existence of an effect of childhood unpredictability on the foraging task. A recent report indicated that adults who retrospectively recalled more stressful events in their childhoods explored less in a foraging environment (54). The discrepancy between this report and the present data could reflect a distinction between data based upon adult recall of childhood events versus reporting of children’s current lived experiences. However, it could also reflect developmental differences. Past studies showed that adults performed at around the optimal level to balance between exploration and exploitation (34), or even under-explore (55), in foraging tasks. However, our sample performed in ways consistent with other studies with young participants (2, 11), suggesting that children tend to explore more than what is optimal. Future studies could examine a continuous age range when individual variations in exploration would manifest in the foraging context. Children’s expectations of predictability could also vary with cultural norms (56); more diverse samples could closely examine these possibilities.

Environmental unpredictability, though currently understudied, constitutes an important dimension of the childhood environment. What might lead a child to perceive unpredictability can consist of ubiquitous everyday occurrences that are subjective, rendering it a difficult dimension of people’s lives to measure, but still one that warrants greater attention in understanding human development (16). Many of the events thought to contribute to perceptions of unpredictability such as caregivers not following through with planned activities and inconsistent responsiveness to children’s requests would not be considered stressors within conventional conceptualizations of childhood adversity. However, these types of unpredictability in caregiving have been theorized to affect the biobehavioral systems associated with infant–caregiver bonding, attachment, and perceptions of safety (57–59). The association between childhood unpredictability and reduced information-directed exploration appears to be robust; in the present study, it was consistent across community samples using both in-person and online methods. These findings reveal the effect of a dimension of childhood experiences on how learning occurs.

## Conclusion

This study found an association between childhood unpredictability and reduced exploration for information, which is accounted for by a habitual preference for familiarity. Exploration occurs in all facets of life, from the discovery of the physical world to formal educational settings. It is also a critical component of socio-emotional learning that sets the stage for social competency and emotional well-being later in life (42). Individuals who have difficulty sampling information from and flexibly responding to their circumstances may be limited by a calcified behavioral repertoire, miss opportunities for deeper learning, develop less sophisticated abilities in emotion reasoning, or be at increased risk for compromised mental health outcomes (35, 60, 61). However, exploiting previous options may be a feature that allows children to cope with unstable environments and confers adaptation to those childhood contexts (62). These insights into the development of decision-making further reveal the myriad ways in which early experiences exert broad effects on the way humans learn from and adapt to their changing circumstances.

## Materials and Methods

### Participants.

Eighty-three 10- to 13-y-olds (M = 11.2, SD = 0.96, 38 female) visited the laboratory to participate in the primary study. They represented a community sample from the Madison, WI metropolitan area and were recruited from a registry of K-12 students in the Madison Metropolitan School District. In terms of race, 67% self-identified as white, 9% as Black, 9% as Hispanic, 4% as mixed race, 5% as Asian/Pacific Islander, and 6% as “Other.” Four participants had missing data about family income because parents chose not to answer the question, and two participants were missing digit span data because of incorrect task administration, leaving 78 adolescents with complete data for analysis.

All parents and children gave informed consent/assent for the study and the University of Wisconsin-Madison Institutional Review Board approved all procedures. At the conclusion of the study, children selected a gift of their choice (worth approximately $20) for participating and parents were paid $25.

### Procedures.

Participants completed two computerized exploration tasks in a counterbalanced order, followed by a test of numeric working memory (digit span task) to examine whether general cognitive abilities may influence the decision-making task performance. In between the tasks, children completed questionnaires about their environments. In a separate room, parents completed questionnaires about parenting practices and family demographics.

#### Measures of explore-exploit decision-making.

##### Horizon task.

We used a bandit-type task that presents participants with an information-seeking problem (37, 63; Fig. 1*A*; see *SI Appendix* for task instructions). Participants played 80 self-paced games in total in randomized order. Each game consists of multiple trials where participants choose between two one-armed bandits that pay out differing point values to maximize their cumulative rewards. Participants were informed that each bandit was relatively consistent in its payoff amount within each game and that one bandit, in general, pays better than the other. After selecting a bandit, participants saw only the points awarded by their chosen bandit. The mean of the left bandit was set to either 40 or 60, and the mean of the right bandit was set to have a relative difference of 4, 8, 12, 20, or 30. Payouts of each trial were sampled from a Gaussian distribution with an SD of 8 and rounded to the nearest integer. Games were counterbalanced on information, reward amount, and whether the left or right bandit was the high mean option. The computer determined the first four selections, which would not count toward the gains. These fixed choices controlled which information participants were exposed to before making their first free choice. In our version of this task, all games presented “unequal information” where there was an imbalance of information about the two bandits. That is, within the first four choices, the computer always selected one bandit three times and the other bandit once. Choosing the bandit with fewer previous payouts (i.e., “high information option”) at the first free choice (fifth trial) was defined as exploration. By doing so, the participant would gain more information about the payoff amounts of the two bandits. After that trial, information on the high-mean option increased as a function of the free-choice trial number, resulting in reward and information confounding each other. As expected, participants learned to choose the bandit with a higher mean payout history and to decrease information seeking as each game progressed (*SI Appendix*, Fig. S1).

Additionally, this task manipulated the horizon, which was the number of trials where one could use the information gained from exploration. Each game was either 5 or 10 choices in length, corresponding to short and long horizons, respectively. We defined strategic exploration as a tendency to use the horizon information to guide exploration—exploring more in long-horizon games compared to short-horizon games.

##### Orchard task.

In this patch foraging task (34, 64; *SI Appendix*), participants spent 14 min harvesting apples in a series of four orchards, each taking the same amount of time (Fig. 1*B*). Participants were told that they should try to collect as many apples as possible within the fixed time frame and that apples would later be converted to points. The apple supply at each tree gradually dwindled with repeated harvests. At each trial, participants chose via key press to either continue harvesting at their current tree (exploit) or move to a different tree (explore). This task poses serial decisions where participants have to infer the expected value of seeking new options that they have not been exposed to. Participants were informed that in some orchards trees were spread out, so it would take longer “travel time” (12 s) to walk to a new tree, and in other orchards, trees were closer together (6 s). Thus, there were differing levels of opportunity costs for moving since participants could not travel and harvest apples at the same time. For that reason, the long-travel-time orchards represented a “poorer” foraging environment, and the short-travel-time orchards represented a “richer” foraging environment. Everything else remains the same across the four orchards. Travel time conditions were counterbalanced in an ABAB/BABA block design. In all orchards, the “harvest” time was 3 s, and the mean depletion rate was 0.88 (each harvest yielded 88% of the apples in the previous harvest). Participants were required to harvest at least once at each tree before continuing. On average, in the short-travel-time (richer), orchards participants saw 20.27 (SD = 6.39) trees and harvested 88 (SD = 5, Min = 75, Max = 97) times, and in the long-travel-time (poorer) orchards they saw 13.72 (SD = 4.35) trees and harvested 73 (SD = 10, Min = 50, Max = 88) times.

The dependent variable, the “exit threshold,” was the average of the last two harvests before moving to a different tree. A high exit threshold indicated a higher exploration rate, while a low exit threshold indicated a lower exploration rate. We excluded the first exit threshold in each block, given that participants did not know whether the travel time was “short” or “long” until they traveled to a new tree. Additionally, we calculated the difference between participants’ exit thresholds and the optimal threshold to measure how well participants did in this task. A positive value means that participants explored more than optimal, and a negative value means that participants explored less than optimal. Following previous research (34), we determined the optimal threshold using the Marginal Value Theorem (65): 6.52 for the short-travel-time environment and 5.31 for the long-travel-time environment.

#### Measures of individual differences.

##### Childhood unpredictability.

Participants completed the QUIC (18), which measures perceived unpredictability across domains over a participant’s lifetime. The scale consists of five subscales: Parental Involvement (9 items), Parental Predictability (12 items), Parental Environment (7 items), Physical Environment (7 items), and Safety and Security (3 items). Examples of these subscales include “My parents were often late to pick me up (e.g., from school, aftercare, or sports),” “At least one of my parents was unpredictable,” “I experienced changes in my custody arrangement,” “I moved frequently,” and “There was a period of time when I often worried that I was not going to have enough food to eat.” Scores on the scale can range from 0 to 38, with a higher score indicating greater exposure to unpredictability in the childhood environment. See *SI Appendix* for more information.

Other individual difference measures used as covariates (i.e., numeric working memory and child anxiety) as well as parent reports of negative events in their children’s lives are described in *SI Appendix*. Family income was assessed using the Pollak & Wolfe poverty measure (66).

### Data Analysis.

Data analyses were carried out in R (R Foundation) and MATLAB (MathWorks). All statistical models in this study controlled for covariates of numeric working memory, family income, and child anxiety. A participant-level random intercept was included in all mixed-effects models, along with task-specific random intercepts for horizon, reward-information conflict (*Horizon task*), and environment type (*Orchard task*). To facilitate the understanding of the effect of predictors in generalized mixed-effects models involving interactions, we reported estimated marginal effects for predicted probabilities of the outcome in each condition when holding the other variables constant. Continuous variables were standardized. For each task, we removed any participants from analysis who showed statistically extreme patterns (>2 SDs above or below the mean) of nearly always exploring or exploiting, as these patterns could reflect a misunderstanding of the task goals. Two outliers were identified for the Horizon task and three for the Orchard task (all were >2 SDs below the mean), indicating an excessive tendency to exploit compared to the rest of the sample. Individuals identified as outliers in one task were included in analyses of the other task. For the Horizon task, we conducted two streams of analyses for the a priori hypothesis regarding the relationship between childhood unpredictability and exploration: one using trial-level behavioral data in its native form and the other leveraging a logistic computational model to estimate individualized parameters for information bonus. Results were consistent in both sets of analyses. Analyses with the computational modeling data are reported in *SI Appendix*. Analysis code, data, and stimuli are available at https://osf.io/5ba43/.

### Replication.

After completing data analyses for the first study, we sought to replicate our findings. The COVID-19 pandemic required us to collect data via an online platform but also afforded the possibility to both compare results across independent samples as well as determine whether effects were robust across in-person and remote data collection contexts.

#### Participants.

Participants, ranging from 10 to 13 y of age (M = 11.2, SD = 1.08, 40 female; 87% identified as white, 3% as Hispanic, 4% as mixed, 6% as Asian/Pacific Islander) were recruited from the same database as the first study as well as Facebook ads and https://childrenhelpingscience.com/. Of the 116 participants who completed the study, 2 had missing data in childhood unpredictability, 5 in the digit span task, and 8 in family income, resulting in 101 participants with complete data. To ensure data quality with remote data collection, we asked participants six comprehension questions about the task instructions (*SI Appendix*) and only included participants who answered at least five questions correctly, resulting in an 83.2% retention rate and a final sample size of 84. All parents and children gave informed consent/assent for the study and the University of Wisconsin—Madison IRB approved all procedures. Children received $20 for their participation.

#### Procedures.

The remote study was hosted on Pavlovia and coded using JsPsych (67). At the beginning of each session, we instructed children to be in a quiet place without the distraction of other electronic devices or other people. All procedures in the remote replication study were identical to the first study with the following exceptions. To shorten the task duration in an effort to maintain children’s interest and attention, we only included the Horizon task in the replication study. Second, we used the Perceived Stress Scale (PSS-10, 68; see *SI Appendix* for scale descriptions) to assess the level of stress that adolescents perceived in their current life instead of clinical measures of anxiety. This was because we would not have been able to intervene in situations where children reported clinical levels of distress given the nature of online testing. Finally, the digit span task was presented visually instead of auditorily.

#### Summary of replication.

Task performance was comparable between the Primary and Replication studies, as described in the *Results* and Table 2, except that children explored slightly less overall in the online Replication study. Individual difference measures were also comparable between the two studies (Table 3) except that digit span scores were higher in the Replication study, likely reflecting the larger capacity of visual versus auditory working memory (69).

**Table 2.** Task performance (Mean and SD) by study

	Primary study	Replication study	*t*-value
Exploration	0.52 (0.09)	0.48 (0.10)	2.29*
Strategic exploration	0.03 (0.10)	0.03 (0.14)	0.08
Reaction time	1.44 (0.58)	1.50 (1.00)	−0.38
Reward maximization	0.82 (0.12)	0.79 (0.12)	1.90
Mean reward gained	55.16 (1.90)	54.02 (1.80)	2.35*

Note: Exploration was the percentage of choosing the more informative options at the first free choice averaging across trials. Strategic exploration was the difference of exploration rate in long-horizon versus short-horizon games. Reaction time was the reaction time in seconds at the first free choice. Reward maximization was the percentage of choosing the bandit with a higher mean payout history at the last trial in long-horizon games. Mean reward gained was rewards gained in all free choices averaging across trials. Two-sample *t* tests were used to compare task performance between the Primary and Replication studies. *P < 0.05, **P < 0.01, ***P < 0.001.

**Table 3.** Individual difference measures (Mean and SD) by study

	Primary study	Replication study	*t*-value
Childhood unpredictability	5.63 (5.05)	6.10 (5.70)	−0.61
Family income	4.29 (1.39)	4.55 (1.30)	−1.12
Digit span	10.85 (3.18)	16.11 (5.50)	−6.72***

Note: Two-sample *t* tests were used to compare individual difference measures between the Primary and Replication studies. *P < 0.05. **P < 0.01. ***P < 0.001.

## Supplementary Material

Appendix 01 (PDF)

## Data Availability

The data and analysis code that support the findings of this study are openly available at https://osf.io/5ba43/ (70). All other data are included in the manuscript and/or *SI Appendix*.
