# Supplementary material for: Childhood unpredictability and the development of exploration
Source: Proc Natl Acad Sci U S A. 2023 Nov 27;120(49):e2303869120. doi: 10.1073/pnas.2303869120 (PMC10710094; doi:10.1073/pnas.2303869120)
Supplement: Supplementary file 1 — Appendix 01 (PDF) [file pnas.2303869120.sapp.pdf]

PNAS

**Supporting Information for**
Childhood Unpredictability and the Development of Exploration

Yuyan Xu,[1] Madeline B. Harms,[1] C. Shawn Green,[1]
Robert Wilson,[2] and Seth D. Pollak[1]

[1]Department of Psychology, University of Wisconsin – Madison
1202 West Johnson Street, Madison WI 53706

[2]Department of Psychology, University of Arizona
1503 E. University Blvd. (Building 68), Tucson, AZ 85721

*Correspondence author: Yuyan Xu, Seth D. Pollak
**Email:** yuyan.xu@wisc.edu, seth.pollak@wisc.edu

**This PDF file includes:**

Supporting text
SI Figures 1 to 3
SI Tables 1 to 4

## Additional Individual Difference Measures

**Numeric Working Memory.** In both studies, adolescents completed the Digit-Span test from the Wechsler Intelligence Scale for Children (WISC-III; 1) as one index of individual differences in general cognitive abilities. Participants were administered both forward and backward span starting from 2 digits, and the task discontinued if children provided incorrect responses to both items in the same digit level. A total score was created by adding the forward and backward span scores, and it was normalized to a standardized score based on participants' age according to the WISC-III scoring manual. Scores on the scale can range from 0 to 30, with a higher score indicating better performance.

**Child Anxiety**. In the Primary Study, thirty-seven participants completed the Multidimensional Anxiety Scale (MASC, 2) and 39 participants completed the Revised Children's Manifest Anxiety Scale (RCMAS-2, 3) for the level of anxiety that they were currently experiencing. In MASC, participants rated how much each of the 39 items is true for them on a four-point Likert scale from "Never" to "Often". This scale captures Social Anxiety (e.g., "I worry about other people laughing at me."), Separation Anxiety (e.g., "I try to stay near my mom or dad."), Harm Avoidance (e.g., "I keep my eyes open for danger."), and Physical Symptoms (e.g., "I'm jumpy."). In RCMAS, participants circled "yes" or "no" for each of the 37 items that measured their Physiological Anxiety (e.g., "My hands feel sweaty."), Worry/oversensitivity (e.g., "My feelings get hurt easily."), and Social Concerns (e.g., "Others seem to do things easier than I am."). Scores were standardized within each measure before we combined them into one variable for all participants in the Primary study.

**Stressful Life Events**. In the Primary Study, parents completed a Life Event Scale to measure their children's exposure to potentially stressful and unstable situations, such as parents' divorce, loss of job, and the death of a relative/friend, in their lives (4). Different from the dimensional approach of stress assessment (e.g., harshness versus unpredictability), this scale provides a primitive retrospective overview of external events and situations that are hypothesized to pose challenges that will lead to difficulties in adaptation. Parents indicated whether each type of event had occurred to their children in the past. A composite score was computed by summing all items.

**Perceived Stress**. In the Replication Study, participants completed the 10-item Perceived Stress Scale (PSS; 5), which measures subjectively experienced stress. Participants' responses to each question (e.g., "How often have you been upset because of something that happened unexpectedly?", "How often have you felt that you were unable to control the important things in your life?") will be on a five-point Likert scale from "Never" (0) to "Very Often" (4). The score range is 0 to 40, with a higher score indicating a higher level of perceived stress.

## Model-based Analysis

**Model.** In addition to behavioral data, we also estimated information-directed exploration using computational parameters. This computational model tracks the participants' reward value estimation for one of the two bandits in each game, $Q_a$ , which is the weighted sum of the expected reward $R_a$ , information $I_a$ , and spatial location $s_a$ . In this equation, $\alpha$ denotes the information bonus and B the side bias. Information-directed exploration was quantified in a model-based manner as the "information bonus" – the additional value given to the more informative option (6).

$$Q_a = R_a + \alpha I_a + B s_a$$

This value estimation $Q_a$ is then translated into a probabilistic choice governed by a decision noise sigma (σ). The model below states that the probability of choosing the left bandit is a softmax function of $\Delta R$ (difference in observed mean reward between the left and right options), $\Delta I$ (difference in information

between left and right options; +1 if left is more informative, i.e. played less in forced trials; -1 if right is more informative), A (information bonus), B (side bias), and σ (decision noise).

$$p(left) = \frac{1}{1 + \exp\left(\frac{\Delta R + A\Delta I + B}{\sigma}\right)}$$

**Results.** We replicated the finding that childhood unpredictability was associated with reduced directed exploration using the same linear regression model reported in the main text, only replacing the empirical percentage of exploration with the computational parameter of information bonus specified above. Results were consistent in the primary (B = -2.1, *SE* = .90, *t*(73) = -2.34, *p* = .02) and the replication study (B = -3.92, *SE* = .90, *t*(73) = -4.41, *p* <.001). The computational modeling approach did not allow us to use trial-level data to examine strategic exploration or the mediating mechanisms. This is because it generates two parameters averaging across all short and long horizon games for each participant. Thus, the number of observations was reduced from 80 to 2 for those analyses, which significantly wiped out between-trial variance and reduced the power. Analysis code, data, and stimuli are available at https://osf.io/5ba43/.

### Additional Mediating Mechanisms for Reduced Exploration in the Horizon Task

We investigated two additional mediating mechanisms beyond temporal discounting and habitual responding using empirical trial-by-trial behavioral data. The first possibility was that unpredictability may lead to more impulsive choices. We regressed the reaction time of the first free choice on childhood unpredictability and task conditions (i.e., horizon, reward-information conflict) in a linear mixed effects model. Results did not support this hypothesis, as there was no significant change in reaction time associated with increasing unpredictability regardless of the horizon (Primary Study: B = -.03, SE = .08, *t* = -.37 *p* = .71; Replication Study: B = .17, SE = .12, *t*(73) = 1.41, *p* = .16). Next, we tested whether unpredictability hindered reward-maximization motivation or ability, which was not supported either. We regressed the percentage of participants choosing the high-payout option at the last choice of the long-horizon games on childhood unpredictability in a generalized mixed-effects model. Unpredictability had no significant effect on the rate of landing at the “correct” option when children had accumulated more information to disambiguate the context (Primary Study: Odds Ratio = .96, *p* = .68, 95% CI: [0.79, 1.16]; Replication Study: Odds Ratio = .92, *p* = .35, 95% CI: [0.78, 1.09]), controlling for reward differential. Thus, there was no evidence that the reduced directed exploration was associated with impulsive actions or deficits in reward maximization.

### Orchard Task Additional Information

In all orchards, the average initial supply on each tree was randomly drawn from a Gaussian distribution with a mean of 10 and an *SD* of 1. Then it gradually dwindled with repeated harvests, with a depletion rate randomly drawn from a Beta distribution (alpha = 14.9, Beta = 2.0). This setup yielded a mean depletion rate of 0.88, meaning repeated harvest would, on average, yield 12% fewer apples than the previous harvest. Participants needed to press a key to make decisions within a 1 second response window right after the brown dot under each tree turns white (see Main Text Fig. 1**b**), or they received a brief warning message, during which they cannot harvest or travel to the next tree. On average, participants received .01 (*SD* = .02) piece of warnings, indicating that their attention was well sustained during the task. In all orchards, the “harvest” time was 3 seconds. Reaction time was not counted into the task duration to ensure that the stay-or-switch decision was the only factor influencing the number of apples obtained. At the end of each orchard, participants saw the number of points they got in that orchard.

**Horizon task instructions and comprehension questions**

***Instructions***

The same instructions were presented to participants in the Primary and the Replication study.

Welcome! Thank you for volunteering for this experiment.

In this game we would like you to choose between two stacks of boxes.

The stacks of boxes look like this.

Every time you choose one stack, the lever will be pulled like this.

And the points you earn will be shown like this. For example, in this case, you chose the left stack this box is giving you 77 points.

Most of the time you will find about the same number of points for all the boxes in a stack, but some may give you a few more or a few less points than others.

For example, the average reward for the stack on the right might be 50 points, but the first box might give us 52 points.

On the second box we might get 56 points.

If we open a third box on the right we might get 45 points this time.

… and so on, such that if we were to play the right bandit 10 times in a row we might see these rewards.

Both bandits will have the same kind of variability and this variability will stay constant throughout the experiment.

On each game, either the left or the right stack will give you more points on average and is the better option to choose for that game.

To make your choice: Press ‘<’ to play the left stack, and Press ‘>’ to play the right stack.

On each game you can tell how many choices you will have by the height of the stacks. For example, when the bandits are 10 boxes high, there are 10 trials in each game.

When the stacks are 5 boxes high there are only 5 trials in the game.

Finally, for the first 4 choices in each game we will tell you which option to play.

There will be a green square inside the box we want you to open and you must press the button to choose this option so you can collect your points and move on the next trial. For example, if you are instructed to choose the left box on the first trial, you will see this.

If you are instructed to choose the right box on the second trial, you will see this.

For the last few choices in each game, you will see two green squares which means you can pick either stack. Each time you see 2 new stacks of boxes on the screen, a new game is starting.

So, to be sure that everything makes sense lets go through some things.

[Comprehension questions will be administered here.]

Press space when you are ready to begin. Good luck!

***Comprehension questions***

In the Primary study, these questions were administered verbally to participants. Experimenters corrected and re-instructed participants who did not provide a reasonable answer to an item. In the Replication study, these questions were presented to participants on the computer screen. Conditional feedback will be provided for wrong choices. Then the question would repeat for a second time to re-check understanding.

1. When it’s your turn to choose, what choice are you making?

- Left or right sets of boxes/bandits

2. What keys are you going to use?

- Left arrow for the left side, right arrow for the right side

3. If you see a box with the number 40 in it, what does that tell you about what numbers are in the other boxes in that stack?

- They will be around 40.

4. If you see a green box only on the left side, that means you have to choose that side. But what does it mean when the green is on both sides?

- My turn to pick between the left side and right side.

5. How are you going to decide what side to pick?

- Reference to goals of task: to get as many points as possible, and/or decide based on the information on each side’s boxes.

**Orchard task instructions**

Before participants started the task, they received task instructions and performed a short practice session where they were presented with both types of foraging environments and practiced pressing the keys for different decisions. This pre-exposure allowed participants to familiarize themselves with the task setup and strategy selection.

Thank you for participating! In this game, you will make choices that can earn you points.

You will be in a series of orchards where you visit trees to harvest apples. You have a limited amount of time in each orchard and you need to decide whether to spend this time harvesting apples at a tree or moving to a brand new tree.

Some trees produce more apples than others and each apple is worth points. Your goal is to earn as many apples as possible.

On each trial you will see a tree. You can decide to harvest apples by pressing the Down arrow key or move to a new tree by pressing the Right arrow key.

Harvesting apples takes up some time but earns you points. However, you will tend to find that the more

times you harvest apples at the same tree, the fewer apples it produces.

Going to a new tree also takes up time but the new tree has a full supply of apples.

When the circle at the bottom of the tree turns white you must decide whether to harvest apples (Down arrow key) or move to a new tree (Right arrow key) You won't have long to make a choice!

If you don't respond in time, you miss a turn. A time-out looks like this.

You will harvest apples in different orchards. Some orchards have spread out trees and it takes longer to reach a new tree. Other orchards are more packed together and it takes less time to reach a tree.

You are now ready to try a short practice session. Remember which keys to use?

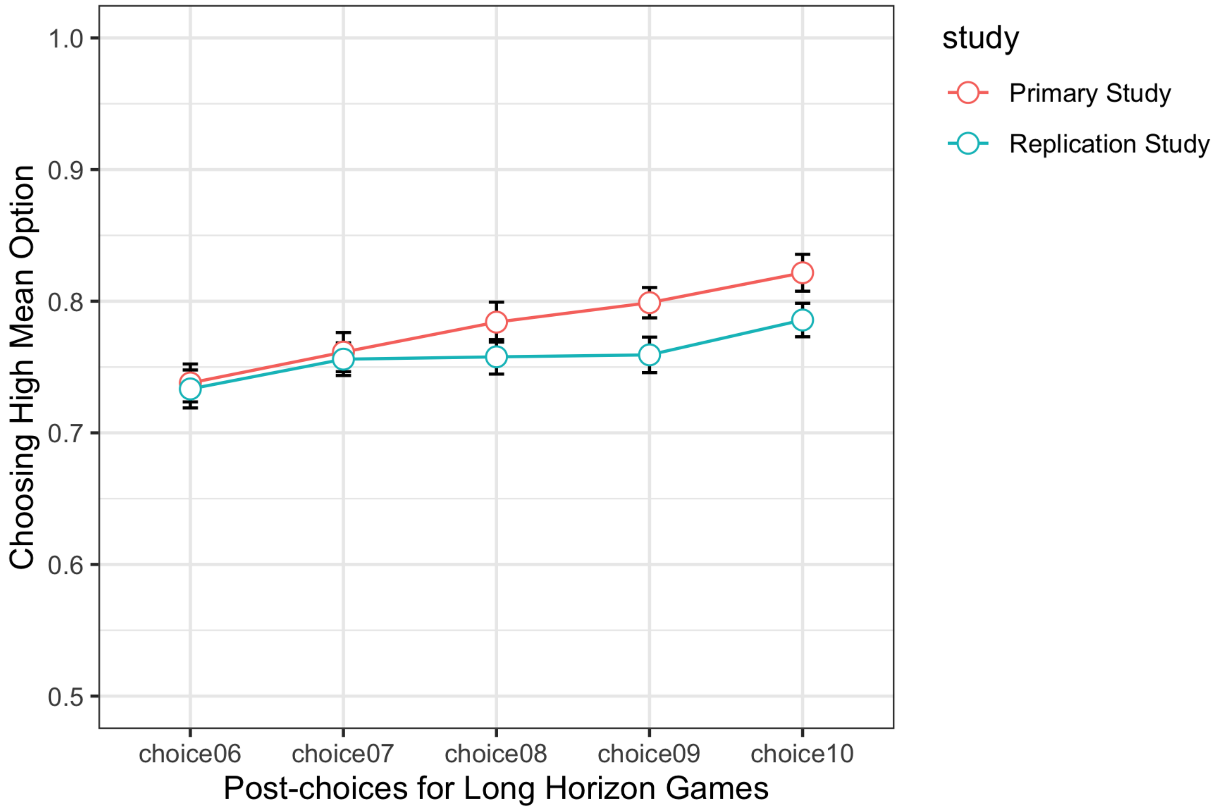


**Fig. S1.** Percentage of choosing the high mean option in post choices for long horizon games.

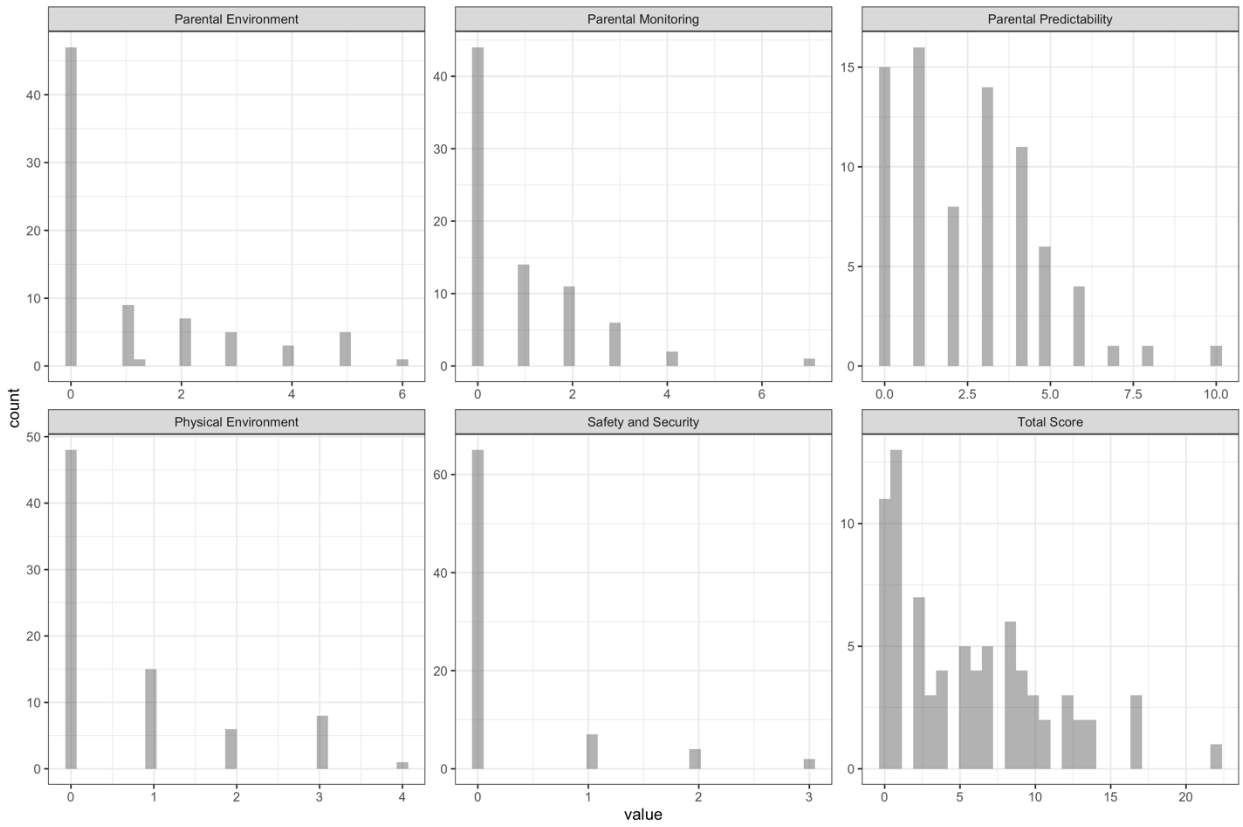


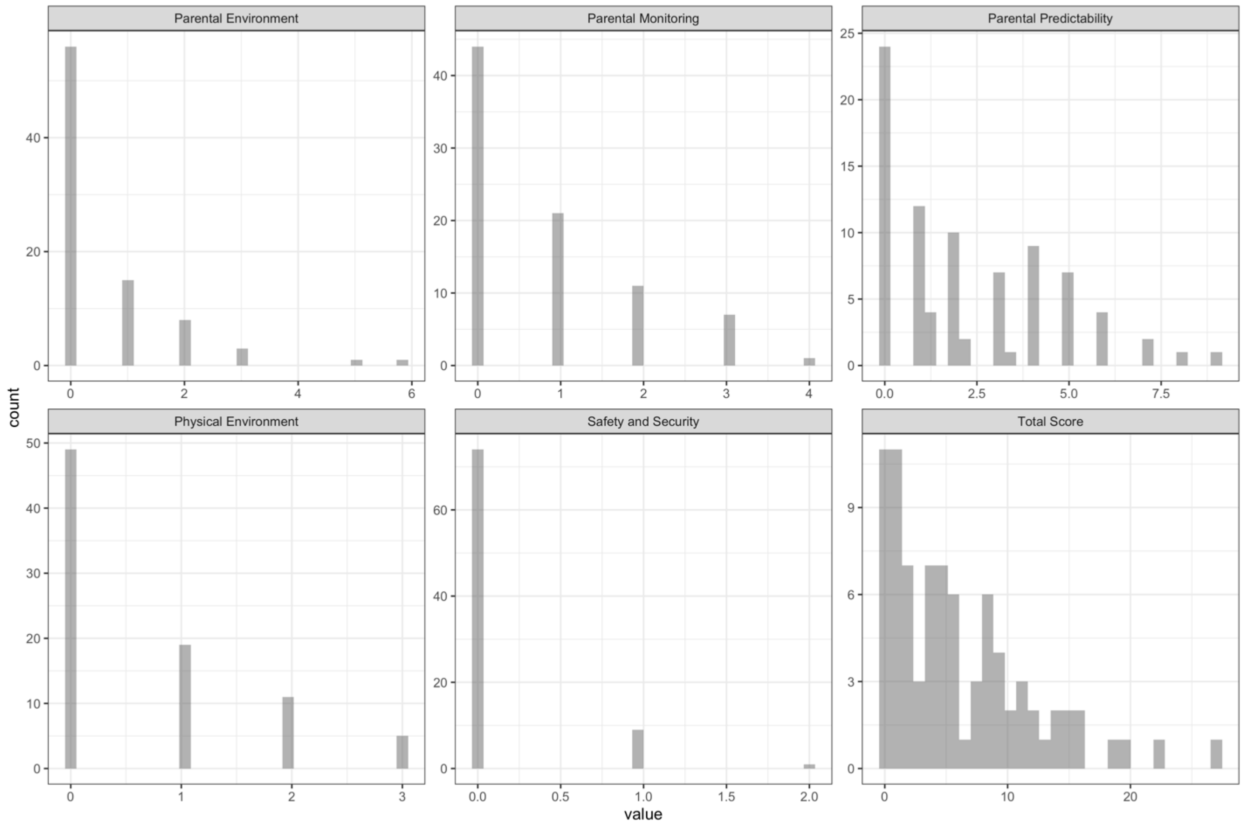


**Fig. S2.** Distribution of subscales and the total score of Questionnaire of Unpredictability in Childhood by Study (top: Primary Study; bottom: Replication Study).

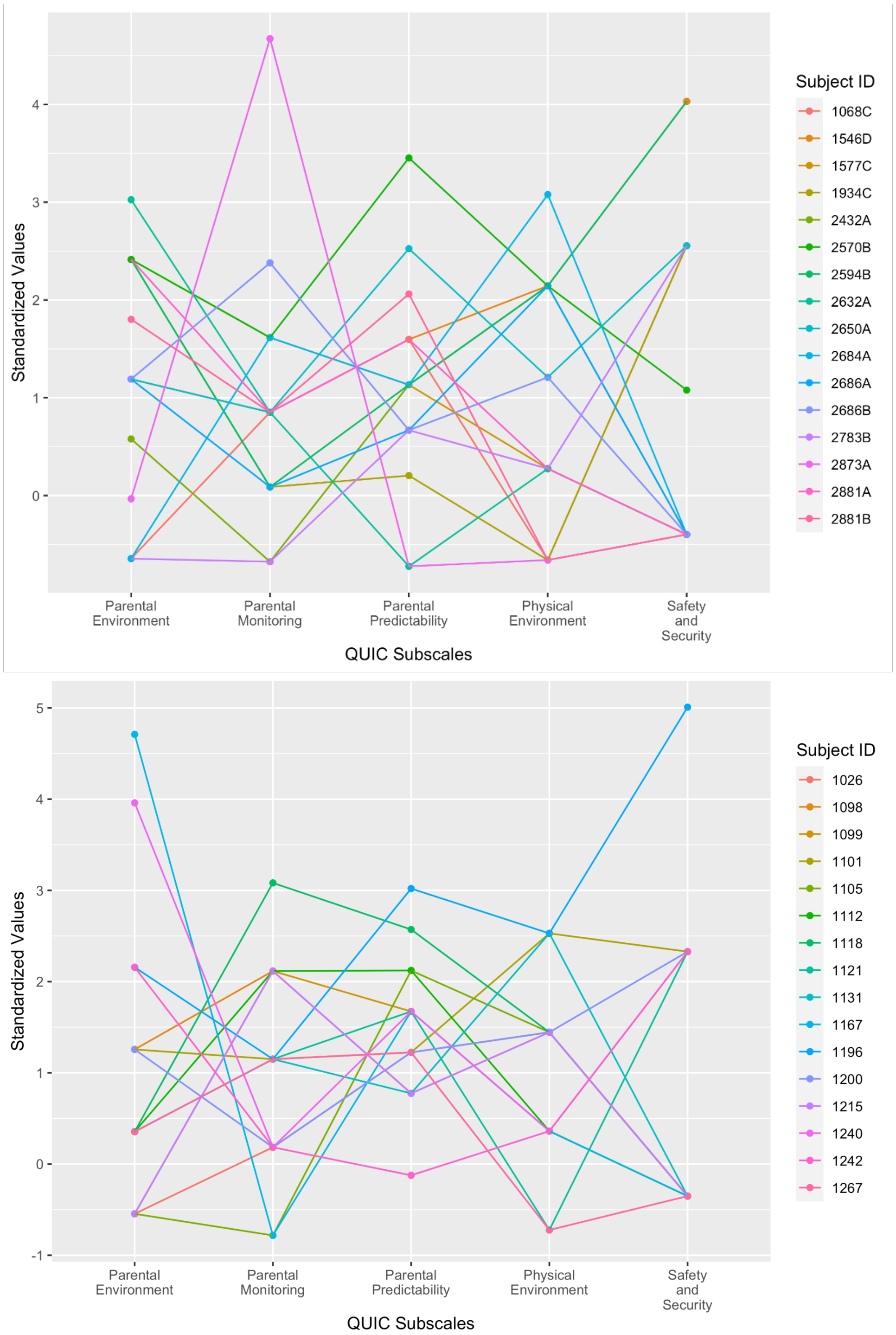


**Fig. S3.** Individual variability in the pattern of responses to each subscale of the Questionnaire of Unpredictability in Childhood for participants who scored in the top 20% in total score in each Study (top: Primary Study; bottom: Replication Study).

**Table S1.** Pearson correlation of individual difference measures in the Primary Study.

| | Childhood unpredictability | Family income | Digit span | Anxiety | Stressful life events |
|---|---|---|---|---|---|
| Childhood unpredictability | -- | | | | |
| Family income | -.53*** | -- | | | |
| Digit span | -.21 | .33** | -- | | |
| Anxiety | .21 | -.06 | -.10 | -- | |
| Stressful life events | .46*** | -.33** | -.25* | .14 | -- |

* $p<.05$. ** $p<.01$. *** $p<.001$.

**Table S2.** Pearson correlation of individual difference measures in the Replication Study.

| | Childhood unpredictability | Family income | Digit span | Perceived stress |
|---|---|---|---|---|
| Childhood unpredictability | -- | | | |
| Family income | .003 | -- | | |
| Digit span | -.02 | .11 | -- | |
| Perceive stress | .47*** | .24* | .01 | -- |

* *p*<.05. ** *p*<.01. *** *p*<.001.

**Table S3.** Pearson correlation of subscales of Questionnaire of Unpredictability in Childhood in the Primary Study.

| | Parental Monitoring | Parental Predictability | Parental Environment | Physical Environment | Safety and Security |
|---|---|---|---|---|---|
| Parental Monitoring | -- | | | | |
| Parental Predictability | 0.38** | -- | | | |
| Parental Environment | 0.32** | 0.53*** | -- | | |
| Physical Environment | 0.23* | 0.47*** | 0.36** | -- | |
| Safety and Security | 0.15 | 0.45*** | 0.36** | 0.27* | -- |

* $p$<.05. ** $p$<.01. *** $p$<.001.

**Table S4.** Pearson correlation of subscales of Questionnaire of Unpredictability in Childhood in the Replication Study.

| | Parental Monitoring | Parental Predictability | Parental Environment | Physical Environment | Safety and Security |
|---|---|---|---|---|---|
| Parental Monitoring | -- | | | | |
| Parental Predictability | 0.47*** | -- | | | |
| Parental Environment | 0.11 | 0.31** | -- | | |
| Physical Environment | 0.34** | 0.41*** | 0.34** | -- | |
| Safety and Security | 0.10 | 0.36** | 0.48*** | 0.27* | -- |

* *p*<.05. ** *p*<.01. *** *p*<.001.

**References**

1. Wechsler, D. (1991). *Wechsler Intelligence Scale for Children (3rd ed.)*. (WISC-III): Manual. San Antonio, TX: The Psychological Corporation.
2. March, J. S., Parker, J. D., Sullivan, K., Stallings, P., & Conners, C. K. (1997). The Multidimensional Anxiety Scale for Children (MASC): factor structure, reliability, and validity. Journal of the American academy of child & adolescent psychiatry, 36(4), 554-565.
3. Reynolds, C., & Richmond, B. O. (1985). Revised children's manifest anxiety scale. Psychological Assessment.
4. Cohen, S., Kessler, R. C., & Gordon, L. U. (1995). Strategies for measuring stress in studies of psychiatric and physical disorders. Measuring stress: A guide for health and social scientists, 28, 3-26.
5. Cohen, S., Kamarck, T., & Mermelstein, R. (1983). A Global Measure of Perceived Stress. Journal of Health and Social Behavior, 24(4), 385–396. https://doi.org/10.2307/2136404
6. Wilson, R. C., Geana, A., White, J. M., Ludvig, E. A., & Cohen, J. D. (2014). Humans use directed and random exploration to solve the explore-exploit dilemma. Journal of Experimental Psychology: General, 143(6), 2074–2081. https://doi.org/10.1037/a0038199
